# Diagnostic utility of various gross thoracoscopic appearances in tuberculous pleural effusion and their correlation with microbiological and histopathological findings – a hospital-based prospective observational cross-sectional study

**DOI:** 10.1097/MS9.0000000000004711

**Published:** 2026-01-21

**Authors:** R Thanisk, Md Arshad Ejazi, Satyadeo Choubey, Manish Shankar, Rakesh Kumar, Bipin Kumar

**Affiliations:** a Pulmonary Medicine, Indira Gandhi Institute of Medical Sciences, Patna, India; b Microbiology, Indira Gandhi Institute of Medical Sciences, Patna, India; c Pathology, Indira Gandhi Institute of Medical Sciences, Patna, India

**Keywords:** cross-sectional study, India, medical thoracoscopy, respiratory medicine, thoracoscopic appearances, tuberculous pleural effusion

## Abstract

**Background:**

Tuberculous pleural effusion (TPE) is a significant global health problem, particularly in high-prevalence regions such as India. Early diagnosis is crucial for effective treatment of this condition. Medical thoracoscopy offers a superior diagnostic yield compared with traditional methods. This study aimed to evaluate various thoracoscopic appearances in TPE, their diagnostic utility, and their correlation with microbiological and histopathological findings.

**Materials and Methods:**

This hospital-based prospective observational cross-sectional study was conducted at a tertiary care teaching hospital in eastern India, from 2023 to 2024. One hundred and twenty-one patients with suspected TPE underwent thoracoscopy, and the findings were classified into 11 categories. Pleural biopsies were examined, and the data were analyzed.

**Results:**

One hundred and three patients were confirmed to have TPE. The most common thoracoscopic findings were easily peelable pleura and adhesions. Significant associations (*P*<0.05) were found between thoracoscopic findings of pustules and positive Cartridge-Based Nucleic Acid Amplification Test results, as well as between easily peelable pleura, multiple variable-sized nodules, and positive mycobacterial culture. Pustules, sago grain nodules, and a few discrete nodules were the most specific findings, with a high positive predictive value. Normal-looking pleura were also observed in TPE. Easily peelable and hard-to-peel pleura were novel findings in our study.

**Conclusion:**

Medical thoracoscopy is a valuable diagnostic tool for TPE exhibiting various gross pleural appearances, and targeted biopsy of various lesions increases the yield of microbiological and histopathological analyses. Findings such as pustules, sago grain nodules, and a few discrete nodules are highly specific for TPE, allowing for the early initiation of antitubercular therapy even before microbiological confirmation.

## Introduction

According to the World Health Organization, the total number of incident cases of tuberculosis (TB) in 2023 was estimated to be 10.8 million. A total of 8.2 million people globally were reported as newly diagnosed with tuberculosis[1]. Extrapulmonary tuberculosis accounts for approximately 15%–20% of all tuberculosis cases in immunocompetent individuals and over 50% in HIV-positive individuals. The lymph nodes are the most frequently affected site, followed by the pleura, though nearly any part of the body can be involved[2]. Tuberculous pleural effusion (TPE) is a significant global health concern, particularly in regions with a high prevalence of tuberculosis. In endemic countries such as India, TPE remains the most common cause of exudative pleural effusions. It accounts for around 38% of cases with exudative pleural effusions[3]. Pleural effusion due to tuberculosis is curable. Hence, it is important to diagnose it as early as possible so that treatment can be initiated and complications prevented.

**HIGHLIGHTS**

- The most common thoracoscopic findings were easily peelable pleura and adhesions.
- Pleural tuberculosis may present even with a normal-looking pleura
- Easily peelable pleura and hard-to-peel pleura are novel findings that are described for the first time in literature.
- Sago grain nodules, pustules, and a few discrete nodules are highly specific for tuberculous pleural effusion
- These thoracoscopic findings may support early empirical treatment in appropriate clinical contexts, pending microbiological and histopathological confirmation.

Medical thoracoscopy is a minimally invasive procedure that offers superior diagnostic yield compared to pleural fluid analysis and closed needle pleural biopsy techniques with diagnostic accuracy approaching almost 100% in differentiating TPE from malignancies and other causes of pleural effusion^[^4-6^]^. In light of the challenges associated with diagnosing TPE, there has been growing interest in utilizing the various gross thoracoscopic appearances of the pleural space as a potential approach for rapid diagnosis^[^7-16^]^. Anti-tuberculous treatment can be started early based on thoracoscopic appearances before microbiological confirmation, which is often the reason for delay in treatment[10]. However, the literature is scarce in this regard. A comprehensive literature review was performed utilizing key databases like PubMed, Embase, and Google Scholar, concentrating on articles published between 2014 and 2024. The search targeted peer-reviewed journal articles and employed pertinent keywords such as tubercular pleural effusion and thoracoscopy. Previous studies on this topic have focused only on a few findings, and no study has examined all the findings in a single study. This study aimed to describe and correlate various thoracoscopic appearances of TPE with microbiological and histopathological findings. This cross-sectional study has been reported in line with the STROCSS guidelines[17].

## Methods

This hospital-based prospective observational cross-sectional study was conducted at a tertiary care teaching hospital in eastern India, over a period of 18 months from May 2023 to October 2024 after obtaining ethical clearance from the Institutional Ethics Committee.

### Inclusion criteria

1. Exudative pleural effusion as per Light’s criteria
2. Lymphocytic predominance in pleural effusion
3. Pleural fluid adenosine deaminase (ADA) level greater than 40 IU/L

### Exclusion criteria

1. Surgically unfit patients (medical conditions like MI, bleeding diathesis, etc.)
2. Seropositivity for HbsAg antigen, HIV, or anti HCV antibodies.
3. Pregnant women

**Sample Size**: The study included 121 participants.

### Sample size calculation

Sensitivity = 94.74%[18]

Prevalence = 15%[18]

Population size = 30 000

Minimum sample size required to conduct the study (n):

Substituting the values:

n = (1.96)^2^ * (0.95) * (0.05)/(0.15) * (0.05)^2^

(At 95% confidence level & 5% margin of error)

n = 121

A total of 121 patients presenting to the outpatient department with undiagnosed exudative pleural effusion after thorough clinical history, physical examination, chest radiology (including chest CT), and diagnostic thoracocentesis who fulfilled the inclusion and exclusion criteria were enrolled in this study and subjected to medical thoracoscopy. Written informed consent was obtained from all participants before their inclusion in this study. No monetary or non-monetary incentivization was used.

### Thoracoscopic procedure

A semi-rigid thoracoscope was used under conscious sedation and local anesthesia. With the patient in the lateral decubitus position with the affected side up, under aseptic precautions, a small skin incision was made at the entry site selected by chest ultrasonography, followed by blunt dissection and trocar-cannula insertion. The thoracoscope was passed through the trocar, the pleural cavity was examined, and the findings were noted. Five to seven biopsies were obtained from abnormal areas in the parietal pleura using the lateral lift and peel technique. A 24 F chest tube was inserted, coupled with an underwater seal bag, and secured with a skin suture. Pleural biopsy samples were sent for histopathological examination, Cartridge-Based Nucleic Acid Amplification Test (CBNAAT), and mycobacterial culture.

### Definition of TPE

TPE was defined using a composite reference standard (CRS) as the gold standard. CRS refers to TB diagnosis based on any of the following criteria:

1. Pleural biopsy histopathological examination showing necrotizing or non-necrotizing granulomatous inflammation or
2. Mycobacterium tuberculosis (MTB) detected on pleural biopsy CBNAAT or
3. Pleural biopsy positive for mycobacterial culture.

### Definition of thoracoscopic appearances

- Normal looking pleura: smooth, grossly normal pleura without other findings.
- Adhesions: fibrinous septa between parietal and visceral pleura.
- Hyperemic pleura: erythematous and edematous parietal pleura.
- Multiple variable-sized nodules: Multiple (>5) nodules of varying sizes (>5 mm).
- Sago-grain nodules: uniform nodules < 5 mm, resembling sago grains, localized or diffusely distributed on parietal pleura.
- Few discrete nodules: single or couple of nodules on parietal pleura (less than five).
- Pustule: pus-filled nodule over parietal pleura.
- Whitish pleural plaques: well-demarcated, raised lesions over parietal pleura.
- Yellowish necrotic patches: peelable patches of caseous necrosis over parietal pleura.
- Easily peelable pleura: parietal pleura manually separable from the chest wall using biopsy forceps with minimal resistance, defined as the ability to peel in one or two attempts using the lateral lift and peel technique.
- Hard to peel pleura: parietal pleura tightly adhered to the chest wall, showing increased resistance to manipulation, defined as requiring more than two attempts to peel using the lateral lift and peel technique.

**Figure 1.**
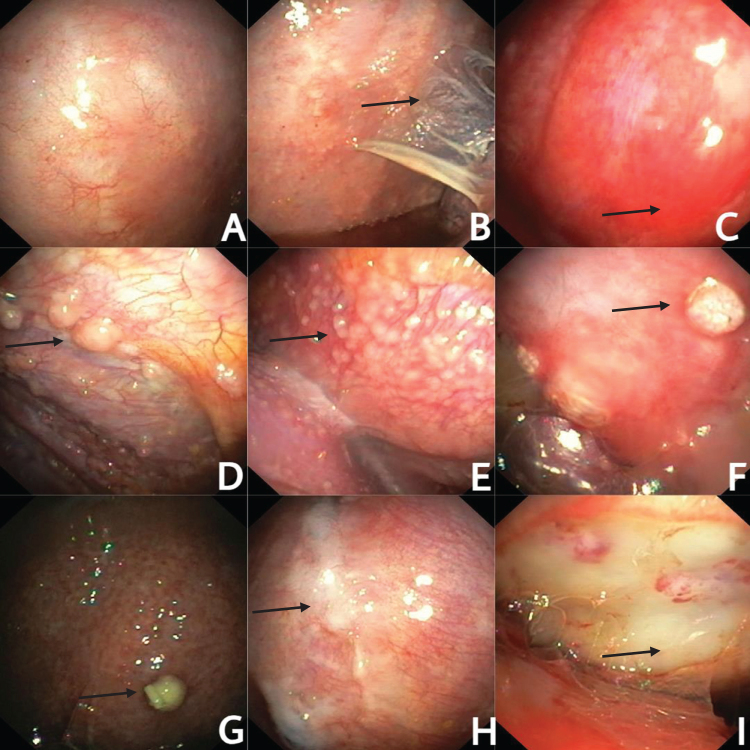
Various thoracoscopic appearances of tuberculous pleural effusion. (A) Normal looking pleura. (B) Adhesions. (C) Hyperemic pleura. (D) Multiple variable-sized nodules. (E) Sago grain nodules. (F) Discrete nodule. (G) Pustule. (H) Whitish pleural plaques. (I) Yellowish necrotic patches.

Figure 1 shows thoracoscopic images of the various appearances described above.

## Statistical analysis

Demographic data, symptoms, side of pleural effusion, pleural characteristics, thoracoscopic findings, and pleural biopsy results were recorded using MS Excel. SPSS v23 (IBM Corp.) was used for the analyses. Descriptive statistics are presented as means/standard deviations and medians/interquartile ranges for continuous variables and frequencies and percentages for categorical variables. The chi-square test was used for group comparisons of categorical data. If the expected frequency in the contingency tables was <5 for >20% of the cells, Fisher’s exact test was used. Linear correlations between continuous variables were explored using Pearson’s correlation (for normally distributed data) or Spearman’s correlation (for non-normal data). Statistical significance was set at *P* < 0.05. The diagnostic accuracy of thoracoscopic appearances was expressed as sensitivity, specificity, and positive and negative predictive values (PPV and NPV).

## Results

Among the 121 patients, 103 were confirmed to have TPE, and 18 were diagnosed with non-tuberculous pleuritis using CRS. A final diagnosis could not be made in 18 patients with non-tuberculous pleuritis. Various parameters, including demographic data, symptoms, pleural fluid characteristics, pleural biopsy microbiological and histopathological findings, and thoracoscopic findings, are presented in Table 1.

**Table 1:** Various parameters and their findings

Parameters	Mean ± SD || Median (IQR) || N (%)
Age (years)	48.93 ± 19.57
Gender	
Male	93 (76.9%)
Female	28 (23.1%)
Dyspnea (yes)	83 (68.6%)
Cough (yes)	91 (75.2%)
Chest pain (yes)	59 (48.8%)
Fever (yes)	70 (57.9%)
Side of effusion	
Right	54 (44.6%)
Left	62 (51.2%)
Bilateral	5 (4.1%)
Pleural fluid	
Sugar	96.45 ± 50.65
Protein	5.15 ± 0.94
LDH	601.19 ± 753.18
TLC	1178.35 ± 1337.63
Neutrophil percentage	10.00 (5.00–20.00)
Lymphocyte percentage	90.00 (80.00–95.00)
ADA	77.90 ± 25.50
Pleural biopsy HPE	
Necrotizing granulomatous inflammation	72 (59.5%)
Non-necrotizing granulomatous inflammation	23 (19.0%)
Chronic non-specific inflammation	16 (13.2%)
Organizing fibrinous pleuritis	8 (6.6%)
Involvement by pulmonary adenocarcinoma	2 (1.7%)
Pleural biopsy CBNAAT: MTB	
Detected	58 (47.9%)
Not detected	63 (52.1%)
Pleural biopsy CBNAAT: Rif Resistance	
Detected	6 (10.3%)
Not detected	52 (89.7%)
Pleural biopsy mycobacterial culture (positive)	54 (44.6%)
Thoracoscopy findings	
Adhesions	64 (52.9%)
Easily peelable pleura	64 (52.9%)
Sago grain nodules	27 (22.3%)
Multiple variable-sized nodules	27 (22.3%)
Pustules	25 (20.7%)
Hyperemic pleura	23 (19.0%)
Whitish pleural plaques	21 (17.4%)
Yellowish necrotic patches	9 (7.4%)
Few discrete nodules	4 (3.3%)
Hard to peel pleura	4 (3.3%)
Normal looking pleura	3 (2.5%)

ADA, adenosine deaminase; CBNAAT, Cartridge-Based Nucleic Acid Amplification Test; HPE, histopathological examination; IQR, interquartile range; MTB, Mycobacterium tuberculosis; N, number; SD, standard deviation; TLC, total leukocyte count; LDH, lactate dehydrogenase.

The association between CBNAAT: MTB detection and thoracoscopic findings, as well as pleural biopsy mycobacterial culture and thoracoscopic findings, is shown in Table 2.

**Table 2:** Association between CBNAAT: MTB detected and thoracoscopy findings and pleural biopsy mycobacterial culture and thoracoscopy findings

Thoracoscopy findings	CBNAAT: MTB		*P* value	Pleural biopsy mycobacterial culture		*P* value
	Detected (n = 58)	Not detected (n = 63)		Positive (n = 54)	Negative (n = 67)	
Adhesions	36 (62.1%)	28 (44.4%)	0.052^b^	33 (61.1%)	31 (46.3%)	0.104^b^
Easily peelable pleura***	36 (62.1%)	28 (44.4%)	0.052^b^	36 (66.7%)	28 (41.8%)	**0.006^b^**
Sago grain nodules	15 (25.9%)	12 (19.0%)	0.368^b^	16 (29.6%)	11 (16.4%)	0.083^b^
Multiple variable-sized nodules***	9 (15.5%)	18 (28.6%)	0.085^b^	6 (11.1%)	21 (31.3%)	**0.008^b^**
Pustules***	21 (36.2%)	4 (6.3%)	**<0.001^b^**	23 (42.6%)	2 (3.0%)	**<0.001^b^**
Hyperemic pleura	11 (19.0%)	12 (19.0%)	0.991^b^	13 (24.1%)	10 (14.9%)	0.202^b^
Whitish pleural plaques	10 (17.2%)	11 (17.5%)	0.975^b^	10 (18.5%)	11 (16.4%)	0.762^b^
Yellowish necrotic patches	3 (5.2%)	6 (9.5%)	0.494^c^	4 (7.4%)	5 (7.5%)	1.000^c^
Few discrete nodules	2 (3.4%)	2 (3.2%)	1.000^c^	2 (3.7%)	2 (3.0%)	1.000^c^
Hard to peel pleura	0 (0.0%)	4 (6.3%)	0.120^c^	0 (0.0%)	4 (6.0%)	0.127^c^
Normal looking pleura	2 (3.4%)	1 (1.6%)	0.607^c^	0 (0.0%)	3 (4.5%)	0.252^c^

*** Significant at *P*<0.05. ^a^t-test; ^b^chi-squared test; ^c^Fisher’s exact test; ^d^Wilcoxon–Mann–Whitney U test; values in bold indicate statistical significance (*P*<0.05).

The diagnostic accuracy of various thoracoscopic appearances in the present study and in previous studies is summarized in Table 3.

**Table 3:** Various thoracoscopic appearances and their diagnostic accuracy

Thoracoscopic findings	References	Sensitivity (%)	Specificity (%)	PPV (%)	NPV (%)
Easily peelable pleura	Current study	51.5 (41–61)	38.9 (17–64)	82.8 (71–91)	12.3 (5–24)
Adhesions	Current study	47.6 (38–58)	16.7 (4–41)	76.6 (64–86)	5.3 (1–15)
Adhesion	Lee *et al*^[^13^]^	71.4	58.4	35.1	86.7
Pleural adhesions	Kong *et al*^[^7^]^	78.95	93.33	98.36	46.67
Sago grain nodules	Current study	25.2 (17–35)	94.4 (73–100)	96.3 (81–100)	18.1 (11–27)
Sago grain appearance	Maturu *et al*^[^14^]^	16.3	99.4	93.7	68.3
Sago-like nodules	Sumalani *et al*^[^16^]^	58.9	92.6	-	-
Sago-like nodules	Lee *et al*^[^13^]^	71.4	93.3	76.9	91.2
Sago-like nodules	Thomas *et al*^[^10^]^	58	89	97	28
Diffuse miliary nodules	Kong *et al*^[^7^]^	64.47	86.67	96.08	32.50
Multiple variable-sized nodules	Current study	25.2 (17–35)	94.4 (73–100)	96.3 (81–100)	18.1 (11–27)
Pustules	Current study	23.3 (16–33)	94.4 (73–100)	96 (80–100)	17.7 (11–27)
Pleural pustule	Maturu *et al*^[^14^]^	17.4	100	100	68.7
Hyperemic pleura	Current study	20.4 (13–29)	88.9 (65–99)	91.3 (72–99)	16.3 (10–25)
Whitish pleural plaques	Current study	15.5 (9–24)	72.2 (47–90)	76.2 (53–92)	13 (7–21)
Yellowish necrotic patches	Current study	5.8 (2–12)	83.3 (59–96)	66.7 (30–93)	13.4 (8–21)
Necrosis	Kong *et al*^[^7^]^	76.32	93.33	98.31	43.75
Few discrete nodules	Current study	3.9 (1–10)	100 (81–100)	100 (40–100)	15.4 (9–23)
Hard-to-peel pleura	Current study	2.9 (1–8)	94.4 (73–100)	75 (19–99)	14.5 (9–22)

NPV, negative predictive value; PPV, positive predictive value.

## Discussion

Differentiating TPE from other causes of exudative pleural effusions, which often have similar clinical manifestations, is challenging. Since the advent of medical thoracoscopy, making a definitive diagnosis of pleural tuberculosis has become easier with direct visualization and targeted biopsies from abnormal areas. In the present study, we conducted medical thoracoscopy in suspected cases of TPE and noted various thoracoscopic appearances, which were classified into 11 entities, as discussed in the methodology section.

The most common thoracoscopic findings were easily peelable pleura (52.9%) and adhesions (52.9%) (Table 1). Similarly, Kong *et al* reported adhesions (78.95%) as their most common finding[7]. The most common pleural biopsy finding on HPE was necrotizing granulomatous inflammation (59.5%). However, no significant association was observed between the thoracoscopic findings and pleural biopsy results.

Pleural biopsy CBNAAT detected MTB in 58 patients (47.9%), of whom six were rifampicin resistant. This is an important advantage of thoracoscopy in TPE, which helps us obtain targeted biopsies from abnormal areas, thereby increasing the yield of pleural biopsy CBNAAT with the added advantage of identifying rifampicin resistance as part of the Universal Drug Susceptibility Testing incorporated in the recent PMDT guidelines in India. The thoracoscopic finding of a “pustule” was significantly associated (*P*<0.05) with positive CBNAAT results (Table 2), in agreement with Maturu *et al*[14]. Hence, pustules should be subjected to CBNAAT as a positive result is more likely.

Two patients tested positive for MTB using CBNAAT, while histopathology revealed malignant lesions identified as pulmonary adenocarcinomas through immunohistochemistry. Mycobacterial cultures were negative in both patients, with no history of TB. Hence, a final diagnosis of TPE with malignancy was made in both cases. This co-occurrence suggests that in endemic areas such as India, where the prevalence of TB is high, patients may have pleural effusion due to both malignancy and coexisting TB.

In this study, 54 patients tested positive for mycobacterial cultures in pleural biopsy. A significant association (*P*<0.05) was observed between pleural biopsy mycobacterial culture and the following thoracoscopic findings: easily peelable pleura, multiple variable-sized nodules, and pustules (Table 2). Easily peelable pleura and pustules are more likely to yield positive culture results. However, multiple variable-sized nodules favored negative mycobacterial culture outcomes.

In this study, adhesions had a sensitivity of 47.6%, a specificity of 16.7%, a PPV of 76.6%, and a NPV of 5.3%. In contrast to our study, Kong *et al*[7] reported a higher sensitivity, specificity, and PPV, whereas Lee *et al*[13] reported a higher NPV (Table 3). Thus, adhesion can be a sensitive finding for detecting TPE; however, its diagnostic utility is limited by low specificity.

The sago grain nodules had a sensitivity, specificity, PPV, and NPV of 25.2 %, 94.4%, 96.3%, and 18.1 %, respectively. Maturu *et al*[14] reported a similar sensitivity, specificity, and PPV but a higher NPV. In contrast, Kong *et al*[7] and Thomas *et al*[10] reported a higher sensitivity with a similar specificity, PPV, and NPV. Although the specificity was similar to that in our study, other findings differed from those of Lee *et al*[13] and Sumalani *et al*[16] (Table 3). These studies suggest that sago grain nodules can be used to diagnose TPE because of their high specificity and PPV.

Pustules had a sensitivity of 23.3%, specificity of 94.4%, PPV of 96.0%, and NPV of 17.7%. Similarly, Maturu *et al*^[^14^]^ reported a sensitivity of 17.4% and specificity and PPV of 100%, but with a higher NPV (68.7%). Thus, pustules can indicate TPE because of their high specificity and PPV. In our study, when both sago grain nodules and pustules were present, the specificity and PPV were 100%. Similarly, the specificity and PPV were 100% when a few discrete nodules were observed, but with low sensitivity and NPV.

Yellowish necrotic patches had a sensitivity of 5.8%, specificity of 83.3%, PPV of 66.7%, and NPV of 13.4% in our study, whereas Kong *et al*[7] reported higher values. Multiple variable-sized nodules exhibited diagnostic performance similar to that of sago grain nodules. Hyperemic pleura had a sensitivity, specificity, PPV, and NPV of 20.4 %, 88.9%, 91.3%, and 16.3 %, respectively. When hyperemia occurred with sago grain nodules, pustules, or multiple variable-sized nodules, the specificity and PPV reached 100%.

Normal-looking pleura were observed in two patients diagnosed with TPE. Similarly, Thomas *et al* and Elfeqy *et al* observed apparently normal looking pleura in some TPE cases^[^10,15^]^. This implies that TB can involve the pleura without gross thoracoscopic abnormalities. Thus, even normal-looking pleurae cannot completely exclude the possibility of tuberculosis.

We observed two novel findings in our study: easily peelable and hard-to-peel pleura. To the best of our knowledge, these findings have rarely been described in the literature. Most cases had easily peelable pleura (52.9%), whereas a few had hard-to-peel pleurae (3.3%). However, the feel of peeling may vary between observers and depends on the experience of the pulmonologist. If the pleura feels easily peelable during biopsy, the probability of TPE being the likely diagnosis is high, as supported by the high PPV (82.8%) in the present study.

Beyond examining the results of earlier research, this study’s primary strength lies in its presentation of new findings, such as easily peelable pleura and hard-to-peel pleura. In addition to describing the various thoracoscopic appearances observed in TPE, this study evaluated their diagnostic utility. This study also explored the correlation between thoracoscopic appearances and microbiological and histopathological findings.

In addition to the single-centric design with a limited sample size of 121 cases, one significant limitation of our study is the lack of research examining how a pulmonologist’s experience affects the visual results of thoracoscopy in TPE. The classification of thoracoscopic appearances is partly subjective, as it depends on the operator’s visual interpretation and experience. Variations in illumination, image capture, and subtle morphological differences may further influence perception. In this study, efforts were made to standardize the assessment by following uniform descriptive terminology and illustrative reference images during evaluation. Such steps aim to enhance consistency and minimize inter-observer variability, although complete objectivity in visual classification remains inherently challenging. Hence, the authors propose future studies to validate the inter-observer variability using blinded ratings or objective scoring. Several thoracoscopic findings may co-occur, which could confound the interpretation of unadjusted associations in addition to other potential confounders (e.g., age, ADA, lymphocyte percentage, etc.). However, multivariate logistic regression was not feasible because of the limited number of non-TPE cases and small overall sample size. This study mainly included highly suspected cases of tuberculosis, as the primary objective was to describe the various thoracoscopic appearances of TPE. Therefore, the study cohort consisted primarily of patients with a high pre-test probability of TPE, resulting in selection bias. Hence, the generalizability of these findings to undiagnosed pleural effusion is limited. These limitations suggest areas for improvement in future research.

## Conclusion

The results of our study suggest that TPE can have various gross appearances during medical thoracoscopy. Even a normal-looking pleura cannot exclude the possibility of tuberculosis. However, experienced pulmonologists can visually diagnose TPE with great confidence. Whenever a pustule is identified, it should be sampled and sent for CBNAAT and mycobacterial culture, as there is a strong likelihood that these tests will produce a positive result. Similarly, when an easily peelable pleura or pustule is identified, it should be subjected to mycobacterial culture, as it is highly likely to produce a positive result. Although TPE can present with a variety of thoracoscopic findings, some findings, such as sago grain nodules, pustules, and a few discrete nodules, can be used to diagnose TPE with confidence because of their high specificity and positive predictive value. These thoracoscopic findings may support early empirical treatment in appropriate clinical contexts pending microbiological and histopathological confirmation.

## Data Availability

Data sharing is not applicable to this article.

## Research registration unique identifying number (UIN)

Not applicable as our study is an observational study.
